# Rearrangement of Retinogeniculate Projection Patterns after Eye-Specific Segregation in Mice

**DOI:** 10.1371/journal.pone.0011001

**Published:** 2010-06-08

**Authors:** Itaru Hayakawa, Hiroshi Kawasaki

**Affiliations:** 1 Department of Molecular and Systems Neurobiology, Graduate School of Medicine, The University of Tokyo, Tokyo, Japan; 2 Global COE Program “Comprehensive Center of Education and Research for Chemical Biology of the Diseases”, The University of Tokyo, Tokyo, Japan; 3 The 21st Century COE Program “Center for Integrated Brain Medical Science”, The University of Tokyo, Tokyo, Japan; 4 PRESTO, Japan Science and Technology Agency, Tokyo, Japan; INSERM U862, France

## Abstract

It has been of interest whether and when the rearrangement of neuronal circuits can be induced after projection patterns are formed during development. Earlier studies using cats reported that the rearrangement of retinogeniculate projections could be induced even after eye-specific segregation has occurred, but detailed and quantitative characterization of this rearrangement has been lacking. Here we delineate the structural changes of retinogeniculate projections in the C57BL/6 mouse in response to monocular enucleation (ME) after eye-specific segregation. When ME was performed after eye-specific segregation, rearrangement of retinogeniculate axons in the dorsal lateral geniculate nucleus (dLGN) was observed within 5 days. Although this rearrangement was observed both along the dorsomedial-ventrolateral and outer-inner axes in the dLGN, it occurred more rapidly along the outer-inner axis. We also examined the critical period for this rearrangement and found that the rearrangement became almost absent by the beginning of the critical period for ocular dominance plasticity in the primary visual cortex. Taken together, our findings serve as a framework for the assessment of phenotypes of genetically altered mouse strains as well as provide insights into the mechanisms underlying the rearrangement of retinogeniculate projections.

## Introduction

Retinogeniculate projections have been widely used for investigating the mechanisms underlying the initial formation and refinement of neuronal circuits during development [1]–[7]. In adult mammals, retinal ganglion cell (RGC) axons from the two eyes are segregated into eye-specific regions in the dorsal lateral geniculate nucleus (dLGN). However, when retinogeniculate projections are initially formed early in development, RGC axons from the two eyes are intermingled in the dLGN. After this initial formation, the refinement of RGC axons proceeds to make distinct eye-specific regions during development before vision [8]–[11]. Once eye-specific patterns of RGC axons are formed, they are relatively stable thereafter [8]–[10], [12].

It has been of great interest whether and when the rearrangement of neuronal circuits can be induced after the initial formation and refinement of neuronal circuits occur during development [13]–[15]. Although previous reports using cats showed that eye-specific projection patterns of RGC axons in the LGN could further be altered by monocular enucleation (ME) even after eye-specific segregation has occurred [16]–[18], several important points have not been addressed. First, detailed and quantitative characterization of this rearrangement is lacking. For example, it is unclear how rapidly rearrangement takes place in response to ME and whether there are specific directions in which retinogeniculate projections preferentially expand. Second, the molecular mechanisms underlying ME-induced rearrangement of retinogeniculate projections are still largely unclear. This is, at least partially, because information about ME-induced rearrangement of retinogeniculate projections in mice is lacking. Because genetically altered mice are commonly used for investigating molecular mechanisms, detailed and quantitative information about ME-induced rearrangement in mice would be extremely valuable. These points prompted us to examine ME-induced anatomical rearrangement in mice quantitatively. In addition, it is intriguing whether or not the rearrangement of retinogeniculate projections can be induced by ME in mice, because earlier studies using cats and rats reported inconsistent results. In contrast to earlier studies using cats, previous reports using rats showed that ME after eye-specific segregation did not alter the distribution patterns of RGC axons in the dLGN [19], [20].

Here, we show that ME after eye-specific segregation induces the rearrangement of eye-specific patterns of RGC axons in the mouse dLGN. Using the commonly used pigmented C57BL/6 strain, we carried out ME after eye-specific segregation and examined the distribution patterns of RGC axons in the mouse dLGN. When ME was performed at postnatal day 10 (P10), the rearrangement of retinogeniculate projections from the remaining eye was observed within 5 days. We further examined the critical period for ME-induced rearrangement of eye-specific patterns and found that the critical period was almost over at P22. Interestingly, we found that the rearrangement along the outer-inner axis (O-I axis), which is the axis perpendicular to the surface of the dLGN [21], [22], occurred more quickly than that along the dorsomedial-ventrolateral axis (DM-VL axis), the axis roughly in parallel to the surface of the dLGN [23]. These results indicate that ME after eye-specific segregation induces the rearrangement of RGC axons in the mouse dLGN. Our findings should be useful for future experiments using mice to examine the molecular mechanisms underlying the rearrangement of RGC axons after eye-specific segregation.

## Results

### ME after eye-specific segregation resulted in the rearrangement of eye-specific patterns in the mouse dLGN

**Figure 1:**
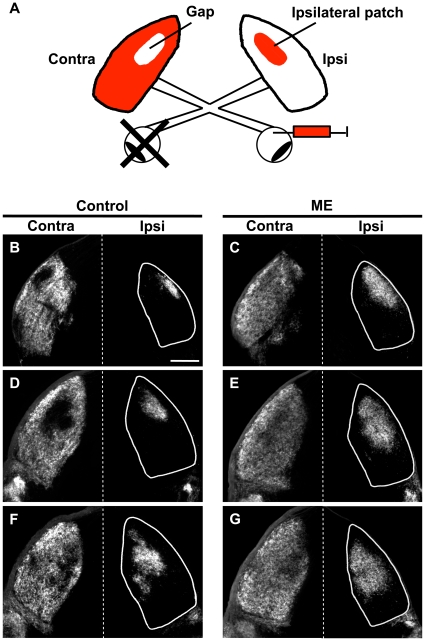
Effects of ME after eye-specific segregation on the remaining retinogeniculate projections in the mouse dLGN. (A) A schematic of experimental procedures. ME was performed at P10, and CTB was injected into the other eye to visualize the remaining RGC axons. Contra and ipsi indicate contralateral and ipsilateral sides to the CTB-injected eye, respectively. The locations of the gap and the ipsilateral patch are also shown. (B–G) Coronal sections of 50 µm thickness were prepared from control (B, D, F) and ME-treated (C, E, G) mice between P35–P37. Representative images of the rostral (B, C), middle (D, E), and caudal (F, G) dLGN are shown. White lines show the boundaries of the dLGN. In each panel, the top is dorsomedial. Scale bar represents 200 µm.

We tested whether RGCs are still capable of changing their projection patterns in the dLGN in response to ME after eye-specific segregation is mostly complete in mice. Because previous studies showed that axonal rearrangement is more robust in younger animals than in older animals in other sensory systems [24], [25], we performed ME on mouse pups at P10, when the eye-specific projection patterns of RGC axons are mostly formed in the dLGN [10], [12]. We then injected Alexa dye-conjugated cholera toxin B subunit (CTB) into the remaining eye to visualize RGC axons in the dLGN, and analyzed the distribution patterns of RGC axons in coronal sections of the dLGN at P35–P37 (Figure 1A). We found that ME at P10 exerted a strong influence on the distribution patterns of RGC axons from the remaining eye in both the ipsilateral and contralateral dLGNs in mice (Figure 1). The ipsilateral retinogeniculate projections occupied a larger area in the dLGN in ME-treated mice than in age-matched control mice (Figure 1B–G, right columns). The difference was evident in coronal sections taken from different parts of the dLGN. Contralateral retinogeniculate projections of ME-treated mice also occupied a larger area and covered almost the entire dLGN (Figure 1B–G, left columns). These results suggest that ME induces the rearrangement of retinogeniculate projections in the mouse dLGN even after eye-specific segregation is mostly complete. Our findings are consistent with earlier studies using cats [16]–[18], but not with those using rats [19], [20].

**Figure 2:**
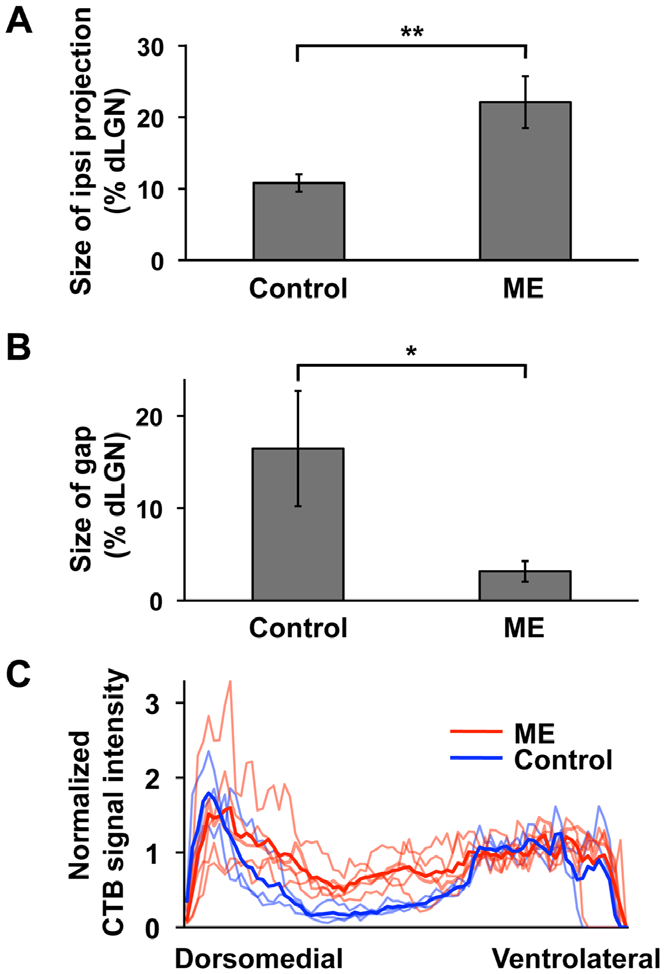
Quantification of the effects of ME after eye-specific segregation on the remaining retinogeniculate projections. ME was performed at P10, and CTB was injected into the other eye. Coronal sections were prepared from ME-treated and control mice between P35–P37. (A) The sizes of the CTB-positive areas relative to those of the dLGNs. The dLGNs ipsilateral to the CTB-injected eye were used. The CTB-positive area was significantly larger in ME-treated mice (n = 5) than that in control mice (n = 3). (**) *P*<0.01, unpaired Student's *t*-test. Error bars represent S.D. (B) The sizes of the gap relative to those of the dLGNs. The gap was significantly smaller in ME-treated mice (n = 4) than that in control mice (n = 4). (*) *P*<0.05, unpaired Welch's *t*-test. Error bars represent S.D. (C) CTB signal intensities within the dLGN contralateral to the CTB-injected eye. CTB signal intensities were plotted against the distance from the dorsomedial tip of the dLGN (see also Figure S2). The signal intensities in the area corresponding to the gap were higher in ME-treated mice (red) than in control mice (blue). Dark lines and light lines represent the averages and data derived from individual dLGNs, respectively.

Next, we quantified ME-induced rearrangement of retinogeniculate projections in the dLGN. The size of the CTB-positive area in the ipsilateral dLGN was measured in the coronal sections 100 µm rostral to the sections containing the largest dLGN area (see Materials and Methods for details). The size of the CTB-positive area relative to that of the dLGN was more than 2-fold larger in the ME-treated group than in the age-matched control group (ME, 22.1±3.6%, n = 5; control, 10.8±1.2%, n = 3; *P*<0.01, unpaired Student's *t*-test) (Figure 2A). Because this difference could result from the expansion of the CTB-positive area and/or the shrinkage of the dLGN in the ME-treated group, we also analyzed the absolute values of the sizes of the CTB-positive area and the dLGN. The size of the CTB-positive area was significantly larger in ME-treated animals than in control animals (ME, 43,673±5,990 µm^2^, n = 5; control, 27,858±3,294 µm^2^, n = 3; *P*<0.05, unpaired Student's *t*-test) (Figure S1A). These results suggest that the area occupied by ipsilateral retinogeniculate projections in the dLGN increases in response to ME in mice. In addition, consistent with a previous report [26], the size of the dLGN ipsilateral to the remaining eye was smaller in ME-treated animals than in control animals (ME, 198,680±15,054 µm^2^, n = 5; control, 257,559±6,849 µm^2^, n = 3; *P*<0.01, unpaired Student's *t*-test) (Figure S1B).

To quantify the retinogeniculate projections in the dLGN contralateral to the remaining eye, we first measured the size of the “gap” [10], [27], [28], which is the region devoid of CTB labeling in the central dLGN (Figure 1A. For the detailed definition of the gap, see Materials and Methods). Consistent with Figure 1, the size of the gap was significantly smaller in ME-treated animals than in control animals (ME, 3.16±1.11%, n = 4; control, 16.46±6.24%, n = 4; *P*<0.05, unpaired Welch's *t*-test) (Figure 2B). We next assessed the distribution patterns of CTB-positive axons within the dLGN (Figure 2C). The CTB fluorescence intensities within a rectangular area (Figure S2, white box) were plotted against the distance from the dorsomedial tip of the dLGN (Figure S2, arrow). We found that ME markedly increased the signal intensities in the area corresponding to the gap. Thus, these results indicate that ME induces the rearrangement of both ipsilateral and contralateral retinogeniculate projections in the mouse dLGN even after eye-specific segregation is mostly complete.

### Time course of the rearrangement of retinogeniculate projections in response to ME

**Figure 3:**
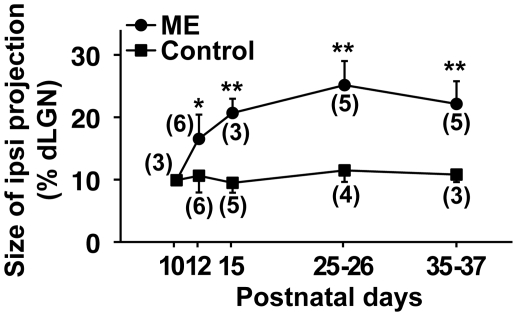
Time course of the rearrangement of retinogeniculate projections induced by ME at P10. ME was performed at P10, and CTB was injected into the other eye. Coronal sections through the dLGN ipsilateral to the CTB-injected eye were prepared at the indicated time points. The sizes of the CTB-positive areas relative to those of the dLGNs are shown. The CTB-positive areas were significantly larger in ME-treated mice than those in age-matched control mice within 2 days after ME. (**) *P*<0.01, (*) *P*<0.05, unpaired Student's *t*-test. Error bars represent S.D. The numbers in parentheses indicate the number of animals.

We next examined the time course of ME-induced rearrangement of retinogeniculate projections. We performed ME at P10 and analyzed the size of the CTB-positive area in the dLGN ipsilateral to the remaining eye at various time points (Figure 3). We found that the size of the CTB-positive area rapidly increased within 5 days after ME and was relatively stable afterwards. Given that eye-specific segregation takes about 10 days to be completed [10], [12], this result suggests that the rearrangement of retinogeniculate projections after eye-specific segregation is a rapid process compared with eye-specific segregation.

### The critical period for ME-induced rearrangement of retinogeniculate projections

**Figure 4:**
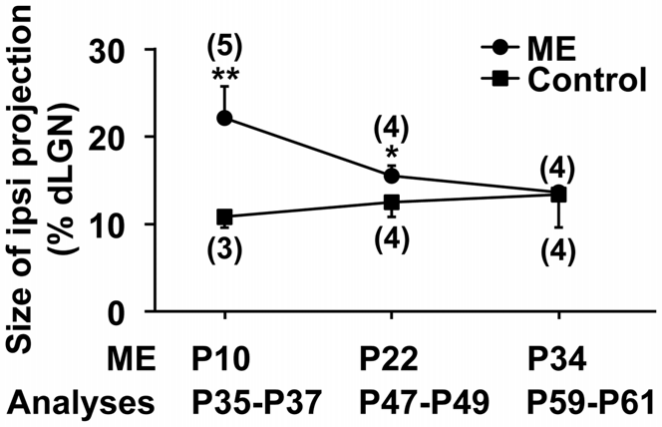
Critical period for ME-induced rearrangement of retinogeniculate projections. ME was performed at the indicated time points, and CTB was injected into the other eye. Coronal sections through the dLGN ipsilateral to the CTB-injected eye were prepared 25–27 days later. The sizes of the CTB-positive areas relative to those of the dLGNs are shown. Note that the CTB-positive areas were significantly larger in mice treated with ME at P10 or at P22 compared with those in control mice. (**) *P*<0.01, (*) *P*<0.05, unpaired Student's *t*-test. The CTB-positive areas did not show significant differences when ME was performed at P34 (*P* = 0.91, unpaired Welch's *t*-test). These results suggest that the critical period for ME-induced rearrangement ends between P22 and P34. Error bars represent S.D. The numbers in parentheses indicate the number of animals.

Although earlier studies using cats showed that ME-induced rearrangement of retinogeniculate projections was less obvious in aged animals [16], [17], these studies did not provide detailed information about the critical period for ME-induced rearrangement. We therefore examined when the critical period for ME-induced rearrangement terminates during development. We carried out ME at either P10, P22, or P34 and measured the size of the CTB-positive area relative to the size of the dLGN 25–27 days later. As shown in Figure 2A, ME at P10 resulted in the expansion of the area occupied by the CTB-positive ipsilateral retinogeniculate projections (Figure 4). In contrast, when ME was performed at P22, there was only a subtle increase in the CTB-positive area (ME, 15.5±1.2%, n = 4; control, 12.5±1.7%, n = 4, *P*<0.05, unpaired Student's *t*-test). ME at P34 did not have an apparent effect upon the size of the CTB-positive area (ME, 13.6±0.5%, n = 4; control, 13.3±3.7%, n = 4, *P* = 0.91, unpaired Welch's *t*-test) (Figure 4). These results suggest that the critical period for ME-induced rearrangement of eye-specific retinogeniculate projection patterns is almost over at P22 in mice. Taken together with earlier physiological studies which showed that the critical period for ocular dominance plasticity in the mouse primary visual cortex is between P19 and P32 [29], [30], our findings indicate that the rearrangement of retinogeniculate projection patterns in the dLGN becomes almost absent by the beginning of the critical period for ocular dominance plasticity in the primary visual cortex.

### The distribution patterns of presynaptic markers in the dLGN after ME

Because it is of interest whether RGC axons giving rise to the expansion of the CTB-positive area contain presynaptic markers, we examined the expression patterns of vesicular glutamate transporter 2 (VGLUT2), which is mainly distributed in RGC axon terminals in the rat dLGN [31], [32]. We performed ME at P10, and coronal sections of the dLGN prepared at P35–P37 were stained with anti-VGLUT2 antibody (Figure S3C, D). In the dLGN ipsilateral to the remaining eye, the VGLUT2-positive area was distributed in the dorsomedial part of the central dLGN, where the remaining RGC axons existed. We found that the extent of the VGLUT2-positive area largely coincided with that of the CTB-positive area (Figure S3C, D). Because the CTB-positive area at P35–P37 had already been expanded in response to ME (Figure 2A), this result suggests that at least some of newly formed retinogeniculate axon terminals express VGLUT2. Consistently, when ME was performed at P34 (i.e. after the end of the critical period), both the CTB-positive area and the VGLUT2-positive area were much smaller than those in mice treated with ME during the critical period (Figure S3A, B).

It should be noted that some VGLUT2-immunoreactive puncta near the optic tract were located outside of the CTB-positive area (Figure S3, arrowheads). Because these puncta did not colocalize with CTB, these VGLUT2-positive puncta appeared to be due to axons derived from brain regions other than the retina. Consistently, a previous report showed similar VGLUT2 immunoreactivities near the optic tract even after binocular enucleation [31]. Because tecto-geniculate axons project predominantly to the outer dLGN [33], and because the superior colliculus expresses VGLUT2 [34], it is reasonable to speculate that these VGLUT2-positive and CTB-negative axons derive from the superior colliculus [35].

We also examined the distribution patterns of VGLUT1. VGLUT1 is mainly expressed in corticogeniculate axon terminals [31], [32], but is not expressed in RGCs [36]. In contrast to VGLUT2, the distribution pattern of VGLUT1 in the dLGN was indistinguishable between ME-treated and control mice, even when ME was performed during the critical period (Figure S4A, B). We further quantified VGLUT1 signal intensities within a rectangular area (Figure S4D, green box) and found that VGLUT1 signal intensities did not change in ME-treated animals (Figure S4D), suggesting that corticogeniculate axons are relatively insensitive to ME. This finding may indicate that ME selectively affects retinogeniculate axons in the dLGN.

### Rapid expansion of the ipsilateral patch along the O-I axis

**Figure 5:**
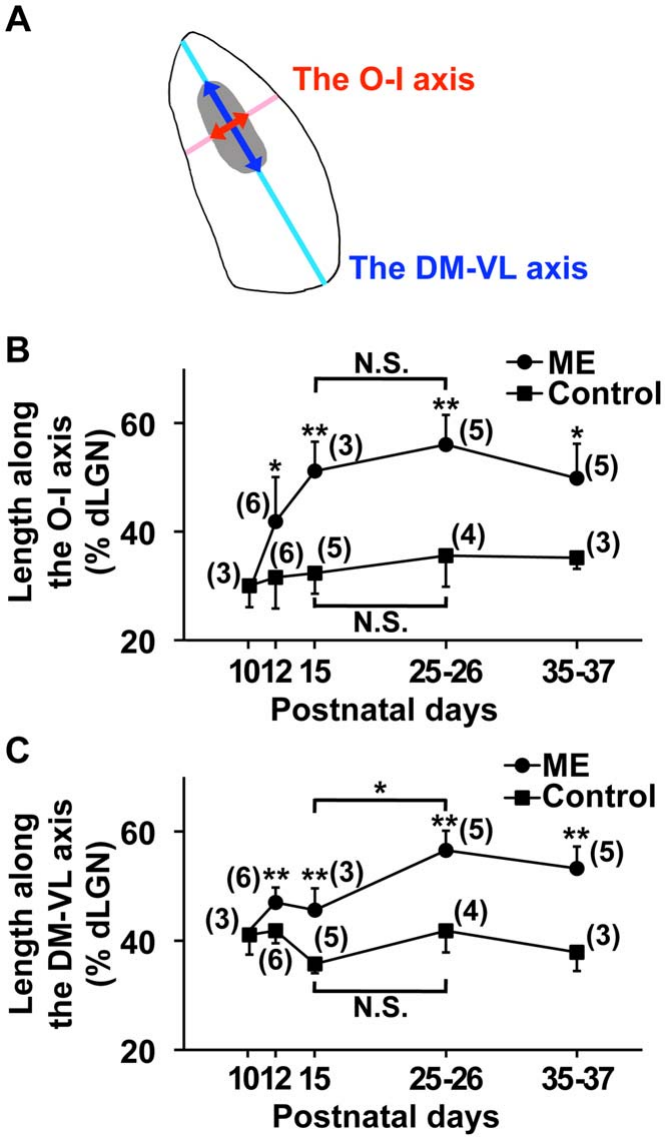
ME-induced rearrangement of retinogeniculate projections along the O-I and DM-VL axes. ME was performed at P10, and CTB was injected into the other eye. Coronal sections through the dLGN ipsilateral to the CTB-injected eye were prepared at the indicated time points, and the ipsilateral patches (gray area) were extracted from the CTB-positive areas. (A) A diagram of the dLGN showing the O-I and DM-VL axes. The lengths of the ipsilateral patch along the DM-VL axis (dark blue arrow) and the O-I axis (dark red arrow) were divided by the lengths of the dLGN along the DM-VL axis (light blue line) and the O-I axis (light red line), respectively (see Figure S5 for details). The values are shown in panels B and C. (B) The lengths of the ipsilateral patch along the O-I axis were significantly larger in ME-treated mice than in age-matched control mice at P15. Note that the lengths did not show significant differences between P15 and P25–P26 in ME-treated mice, suggesting that the expansion reached its maximum within 5 days. (**) *P*<0.01, (*) *P*<0.05, (N.S.) not significantly different (*P*≥0.05), unpaired Student's *t*-test for comparisons between ME-treated and control mice, and Tukey-Kramer test for comparisons between different ages within either the ME-treated group or the control mouse group. Error bars represent S.D. The numbers in parentheses indicate the number of animals. (C) The lengths of the ipsilateral patch along the DM-VL axis were significantly larger in ME-treated mice than in age-matched control mice. In contrast to the lengths along the O-I axis, the length along the DM-VL axis in ME-treated mice at P25–P26 was significantly larger than that at P15. (**) *P*<0.01, (*) *P*<0.05, (N.S.) not significantly different (*P*≥0.05), unpaired Student's *t*-test for comparisons between ME-treated and control mice, and Tukey-Kramer test for comparisons between different ages within either the ME-treated group or the control mouse group. Error bars represent S.D. The numbers in parentheses indicate the number of animals. These results suggest that the expansion of the ipsilateral patch along the DM-VL axis is relatively slow compared with that along the O-I axis.

We next addressed whether retinogeniculate projections in the dLGN preferentially expand in any specific directions in response to ME. We examined the effect of ME along two axes: the DM-VL axis, which is roughly parallel to the surface of the dLGN, and the O-I axis, which is perpendicular to the surface of the dLGN (Figure 5A, Figure S5. See Materials and Methods for details). Previous reports showed that the former roughly corresponds to the nasotemporal mapping axis of the retina [23]. Recent reports have suggested that the latter conveys qualitatively different information from functionally distinct RGC subtypes. For example, a subset of RGCs specialized for detecting posterior motion of objects was reported to send their axons to the outer third of the dLGN, while another subset with different electrophysiological properties projects to inner core of the dLGN [21], [37], [38].

These findings led us to analyze ME-induced rearrangement along these two axes separately. We performed ME at P10 and measured the lengths of the ipsilateral patch along the two axes at the indicated time points (Figure 5B, C). We defined the ipsilateral patch as the CTB-positive areas whose pixel sizes were more than 150 (Figure 1A. See Materials and Methods for details) [39], [40]. Although the ipsilateral patch significantly expanded along both the DM-VL and O-I axes, there was an interesting difference between the two axes. We found that the ipsilateral patch expanded more rapidly along the O-I axis than along the DM-VL axis (Figure 5B, C). The expansion of the ipsilateral patch along the O-I axis was evident within 5 days after ME and was relatively stable thereafter (Figure 5B). In contrast, the expansion of the ipsilateral patch along the DM-VL axis was moderate at P15 and reached its maximum by P25–P26 (Figure 5C). Currently, the reason for this difference is unclear, but these results seem to imply some mechanistic differences of axonal rearrangement along these two axes.

### The involvement of retinal activity in the rearrangement of retinogeniculate projections

**Figure 6:**
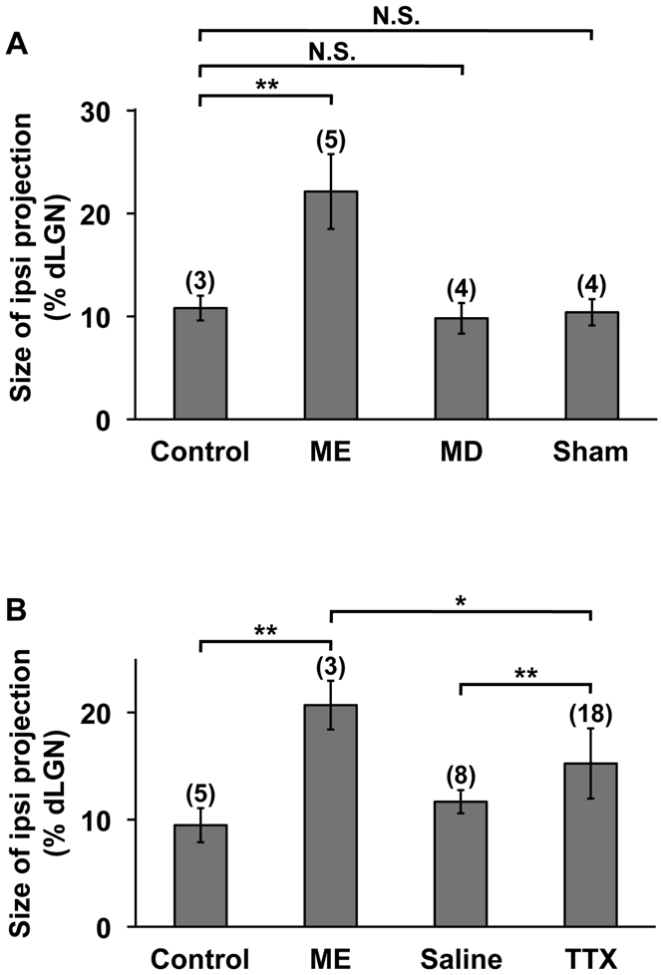
The effects of MD and TTX on retinogeniculate projections. (A) The effect of MD on retinogeniculate projections. P10 pups were treated with or without either ME, MD or anesthesia alone (sham). CTB was injected into the intact eye, and coronal sections of the dLGN ipsilateral to the CTB-injected eye were prepared at P35–P37. The sizes of the CTB-positive areas relative to those of the dLGNs were measured. In contrast to ME, MD did not significantly affect the size of the CTB-positive area. The data of control and ME-treated animals are the same as those in Figure 2A. (B) The effect of TTX on retinogeniculate projections. Pups were treated monocularly with TTX or saline from P10 to P14. CTB was injected into the other eye, and coronal sections of the dLGN ipsilateral to the CTB-injected eye were prepared at P15. The sizes of the CTB-positive areas relative to those of the dLGNs were measured. The data of control and ME-treated animals are the same as those in Figure 3. (**) *P*<0.01, (*) *P*<0.05, (N.S.) not significantly different (*P*≥0.05), unpaired Student's *t*-test or unpaired Welch's *t*-test (see Materials and Methods). Error bars represent S.D. The numbers in parentheses indicate the number of animals.

It seemed possible that the effect of ME on the remaining RGC axons was mediated by the imbalance of visual inputs between the two eyes. Conversely, the effect of ME could have been entirely mediated by activity-independent mechanisms. Because monocular deprivation (MD) results in the imbalance of visual inputs between the two eyes and is known to trigger alterations in the morphology of RGC axons in cats and primates [41], [42], we performed MD by unilateral eyelid suture at P10, which is before natural eye-opening. We examined ipsilateral retinogeniculate projections derived from the non-deprived eye at P35–P37 and found that MD had no significant effect on the size of the CTB-positive area (MD, 9.8±1.5%, n = 4; control, 10.8±1.2%, n = 3; *P* = 0.46, unpaired Student's *t*-test) (Figure 6A). This result suggests that the imbalance of visual inputs between the two eyes is not sufficient for changing RGC projection patterns in the mouse dLGN after eye-specific segregation is mostly complete.

It remained possible, however, that the MD-induced imbalance of RGC activities was not strong enough to trigger the rearrangement, but a stronger imbalance might be sufficient. Indeed, previous reports showed that visual stimuli were able to activate LGN neurons through unopened eyelids [43] and that spontaneous retinal activity was still present during this period [44]. In order to suppress RGC activity, we injected tetrodotoxin (TTX) into one eye from P10 to P14. *In situ* hybridization using the primary visual cortex (V1, Figure S6A) showed that the expression of the immediate early genes *Arc* and *c-fos* in the monocular regions of V1 was markedly suppressed by TTX treatment (Figure S6B, C), as reported previously [45], [46]. When we examined CTB-positive RGC axons derived from the intact eye (i.e. the eye without TTX or saline treatment), we found that ipsilateral retinogeniculate projections occupied a significantly larger area in the dLGN in TTX-treated mice than in saline-treated control mice (TTX, 15.2±3.3%, n = 18; saline, 11.7±1.1%, n = 8; *P*<0.01, unpaired Welch's *t*-test) (Figure 6B). This result suggests that the suppression of retinal activity in one eye results in the rearrangement of retinogeniculate projections derived from the other eye. Interestingly, although TTX induced the rearrangement of retinogeniculate projections, the effect of TTX was weaker than that of ME. The size of CTB-positive area in TTX-treated animals was significantly smaller than that in ME-treated animals (TTX, 15.2±3.3%, n = 18; ME, 20.7±2.3%, n = 3; *P*<0.05, unpaired Student's *t*-test) (Figure 6B). The reason why the effect of TTX was weaker than that of ME could be that retinal activity is not the sole mediator of the effect of ME. On the other hand, we cannot exclude the possibility that the suppression of retinal activity by TTX was not complete, even though the expression of *Arc* and *c-fos* were markedly suppressed in V1 (Figure S6B, C).

## Discussion

We have shown that ME after eye-specific segregation induces the rearrangement of retinogeniculate projections in the mouse dLGN. When ME was performed at P10, the distribution patterns of RGC axons derived from the remaining eye changed significantly within 5 days. The rearrangement was observed both along the DM-VL axis and along the O-I axis, but the rearrangement occurred more rapidly along the latter than along the former. We further examined the critical period for ME-induced rearrangement and found that the critical period was almost over at P22.

It has been of great interest whether and when the rearrangement of neuronal circuits occurs after initial projection patterns are formed [13]–[15]. Our results clearly showed that ME induces the rearrangement of retinogeniculate projections in the mouse dLGN even after eye-specific segregation is mostly complete, and that there is a critical period for this rearrangement. Because our findings are consistent with the results obtained using the cat LGN [16]–[18], our findings also indicate that ME-induced rearrangement of RGC axons in the LGN is not exclusive to carnivores. Because mice and cats are phylogenetically far in mammals, belonging to the distinct clades Euarchontoglires and Laurasiatheria, respectively, our results imply that ME-induced rearrangement of RGC axons after eye-specific segregation could be widely observed in mammals.

Earlier studies using cats showed that ME resulted in the rearrangement of retinogeniculate projections in the LGN [16]–[18]. When ME was performed after the completion of eye-specific segregation, a significant number of RGC axons grew across laminar borders in the LGN and passed from one lamina into the next [16], [17]. In these previous reports, however, quantitative information about ME-induced rearrangement was insufficient to answer a number of questions. It was unclear how rapidly the rearrangement of retinogeniculate projections occurs in response to ME, to what extent retinogeniculate projections can expand in the dLGN, and whether there are any preferential directions in which RGC axons extend in response to ME. These points should be important for evaluating the results of future experiments using genetic or pharmacological manipulations. Our quantitative findings answer these questions and could contribute to future investigations into the molecular mechanisms underlying axonal rearrangement in the dLGN using mice.

Our results showed that monocular TTX treatment led to the rearrangement of retinogeniculate projections derived from the untreated eye, suggesting that the imbalance of retinal activity results in the rearrangement of retinogeniculate projections. It would be interesting to uncover the molecules mediating the effect of TTX on this rearrangement. Similar to the ME-induced rearrangement of RGC axons described here, the segregation of RGC axons into ON and OFF sublaminae in the ferret LGN takes place after eye-specific segregation [47], and this segregation is mediated by activity-dependent mechanisms [48], [49]. Because both of these two distinct events involving retinogeniculate projections occur soon after eye-specific segregation is mostly complete and are at least partially dependent on retinal activity, they could share similar molecular mechanisms. It is possible that the molecules mediating ON/OFF segregation in the ferret LGN such as NMDA receptors, nitric oxide synthase, soluble guanylyl cyclase, and calcineurin are also involved in ME-induced rearrangement of eye-specific patterns in the mouse dLGN [48], [50]–[53]. Our results also showed that the effect of TTX treatment on the rearrangement of retinogeniculate projections was weaker than that of ME. This raised the possibility that mechanisms distinct from the imbalance of retinal activity are also involved in the effect of ME. Because trophic factors such as brain-derived neurotrophic factor (BDNF) are released from RGC axon terminals [54], [55], the lack of trophic factors could mediate the effect of ME.

We found that the ipsilateral patch expanded more rapidly along the O-I axis than along the DM-VL axis. Interestingly, previous reports showed that in response to ME, RGC axons invaded the binocular segment more densely than the monocular segment in the cat LGN [16], [17], [56]. Taken together, these results suggest that the rearrangement of RGC axons in the dLGN preferentially occurs along the O-I axis in both mice and cats. This raises the possibility that the mechanisms underlying the rearrangements along the O-I axis and along the DM-VL axis are different. Indeed, a previous report using cats demonstrated that the rearrangements of retinogeniculate projections toward the binocular segment and toward the monocular segment involved distinct types of axonal rearrangements [56]. It was reported that the rearrangement toward the binocular segment resulted from the elongation of RGC axons within the LGN, whereas that toward the monocular segment involved aberrant axons that originated directly from the optic tract [56]. It would be intriguing to examine whether these two distinct mechanisms observed in cats are also involved in ME-induced rearrangement along the O-I and DM-VL axes in mice. Another possibility would be the involvement of axon guidance molecules. Because ephrin/Eph signaling determines the topographic distributions of RGC axons in the dLGN during early development [57], ephrin/Eph signaling could underlie the spatial restriction of the rearrangement of RGC axons along the DM-VL axis. The other possible mechanism mediating the rearrangement of retinogeniculate projections by ME could be misrouting and/or collateral sprouting of RGC axons at the optic chiasm [19], [26], [58], [59]. Previous reports showed that when ME was performed just after birth, ipsilateral projections derived from the remaining eye expanded, and the number of ipsilaterally projecting RGCs in the remaining eye increased. It was suggested that this effect of neonatal ME could be due to misrouting and/or collateral sprouting of RGC axons at the optic chiasm [19], [26], [58], [59]. Morphological investigations of single retinogeniculate axons in mice would be helpful to address these possibilities.

In this article, we showed comprehensive information about ME-induced rearrangement of retinogeniculate projections in the mouse dLGN after eye-specific segregation. Our quantitative findings should contribute to future molecular investigations about the extent, time course, preferred directions and the critical period termination of ME-induced rearrangement of retinogeniculate projections.

## Materials and Methods

### Ethics statement

All experiments were performed in accordance with protocols approved by the animal experiment committee at the University of Tokyo.

### Animals and surgical procedures

C57BL/6 mice (*Mus musculus*) were purchased from Japan SLC (Hamamatsu, Japan). The day of birth was counted as P0. All mice were reared on a normal 12 h light/dark schedule. ME experiments were carried out as described previously with slight modifications [60]. After mice were anesthetized with isoflurane, the left eye was removed, and pieces of Gelform (Pfizer, New York, NY, USA) were inserted into the cavity. Eyelids were trimmed and sutured. In most of the animals, a drop of Vetbond (3M, St. Paul, MN, USA) was put on sutured eyelids to prevent reopening of the eyelids. For intraocular injections of TTX, animals were anesthetized with isoflurane, and then 0.5 mM TTX solution (0.1 µl) (Alomone labs, Jerusalem, Israel) or saline was injected into the vitreous humor via a glass capillary tube attached to a Hamilton microsyringe at a rate of 0.5 µl/min. TTX injections were performed once a day from P10 to P14. Samples were taken 24 h after the final injection. For MD experiments, after mice were anesthetized with isoflurane, eyelids were trimmed and sutured. Vetbond was used to prevent reopening of the eyelids. The animals were checked for reopening of the eyelids before sampling, and those with reopened eyelids were discarded. For sham operations, animals were anesthetized with isoflurane for 15 min, which was enough time to carry out ME or MD. For sampling, mice were deeply anesthetized with pentobarbital and transcardially perfused with ice-cold phosphate-buffered saline (PBS) containing 4% paraformaldehyde (PFA). Brains were dissected out, post-fixed overnight in 4% PFA/PBS at 4°C, cryoprotected two overnights in 30% sucrose/PBS, and embedded in OCT compound (Sakura Finetek, Torrance, CA, USA).

To induce the expression of immediate early genes in the visual cortex, mice were put in darkness overnight and then returned to a lighted environment in alert condition. After stimulated with light for 30–60 min, mice were deeply anesthetized with pentobarbital. Brains were removed, frozen in OCT compound and sectioned at 14 µm for *in situ* hybridization.

### Visualization of RGC axons in the dLGN

Labeling of RGC axons was performed a few days before sampling as described previously [61]. Alexa555-conjugated CTB (Molecular Probes, Eugene, OR, USA) was dissolved in saline containing 0.2% dimethyl sulfoxide to make a 0.5% stock. Mice were anesthetized with isoflurane, and the CTB solution (2–5 µl) was injected into the vitreous humor of the right eye with a 33 gauge needle.

### *In situ* hybridization

The plasmid used to generate the mouse *Arc* probe (Genbank accession number NM_018790.2) was a gift from Dr. Paul F. Worley (Johns Hopkins University) [62]. The Genbank accession number of mouse *c-fos* used here was AK154418. *In situ* hybridization was performed as described previously with slight modifications [60]. Sections prepared from fresh-frozen tissues were treated with 4% paraformaldehyde for 10 min and 0.25% acetic anhydride for 10 min. After prehybridization, the sections were incubated overnight at 58°C with digoxigenin-labeled RNA probes diluted in hybridization buffer (50% formamide, 5× SSC, 5× Denhardt's solution, 0.3 mg/ml yeast RNA, 0.1 mg/ml herring sperm DNA, and 1 mM dithiothreitol). The sections were then incubated with alkaline phosphatase-conjugated anti-digoxigenin antibody (Roche, Indianapolis, IN, USA) and were visualized using NBT/BCIP as substrates. In some experiments, to visualize cortical layers, the sections were incubated with 1 µg/ml Hoechst 33342 after *in situ* hybridization. Experiments were repeated at least three times using different animals and gave consistent results.

### Immunohistochemistry

Immunohistochemistry was performed as described previously with slight modifications [63]. For VGLUT2 immunostaining, 30 µm sections were prepared using a cryostat and kept in PBS containing 0.01% sodium azide at 4°C until use. The sections were permeabilized with 0.1% Triton X-100 in PBS and incubated overnight with anti-VGLUT2 antibody (rabbit polyclonal, Synaptic Systems, Goettingen, Germany). After incubation with Alexa488-conjugated secondary antibody and 1 µg/ml Hoechst 33342, the sections were washed and mounted.

For VGLUT1 immunostaining, 30 µm sections were prepared using a cryostat and kept in PBS containing 0.01% sodium azide at 4°C until use. The sections were permeabilized with 0.5% Triton X-100 in PBS and incubated overnight with anti-VGLUT1 antibody (rabbit polyclonal, Synaptic Systems, Goettingen, Germany). After incubation with Alexa488-conjugated secondary antibody and 1 µg/ml Hoechst 33342, the sections were washed and mounted. Experiments were repeated at least three times using different animals and gave consistent results.

### Quantification

Epifluorescence images were acquired using a CCD camera (AxioCam HRc, Zeiss) attached to an uplight Zeiss Axioimager A1 microscope, and ImageJ software (NIH) was used for quantitative analyses. All image analyses were done blind. For quantifying the CTB-positive areas in the dLGN, serial coronal sections of 50 µm thickness through the dLGN were cut on a cryostat and photographed. Each pixel in images used for quantification corresponded to 1.00 µm^2^. In each section, the boundaries of the dLGN were delineated using either a DIC image, a CTB image or a bright-field image taken with slanted illumination [64]. The area within the boundaries was defined as the size of the dLGN.

Among each serial series of sections, the section 100 µm rostral to the section containing the largest dLGN area was used to analyze retinogeniculate projections. The background fluorescent signal intensity, which was determined as the average signal intensity of 40,000 pixels ventromedial to the dLGN, was subtracted from the CTB image.

For quantifying the areas occupied by ipsilateral retinogeniculate projections, thresholds (Ti) were determined as follows:
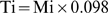
where Mi was the maximum signal intensity in five consecutive sections, the third of which was the section containing the largest dLGN area. The thresholds used here resulted in a clear distinction between signal and residual background fluorescence. The size of the area whose signal intensities were more than Ti was measured and used as the size of ipsilateral retinogeniculate projections.

For quantifying the areas occupied by contralateral retinogeniculate projections, thresholds (Tc) were determined as follows:
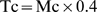
where Mc was the average signal intensity of 10,000 pixels in the ventrolateral monocular segment of the dLGN. The areas whose signal intensities were less than Tc consisted of two kinds of regions: the “gap,” which is the CTB-negative area in the central dLGN (Figure 1A) [10], [27], [28], and the regions that are connected with the contours of the dLGN. In order to measure the size of the gap, the latter was excluded using the flood-fill tool of ImageJ (Figure 2B). The sections whose gap was connected to the contours of the dLGN were discarded.

To quantify CTB fluorescence intensities within the dLGN, a rectangular area (Figure S2, white box), which was 40 pixels wide and which traversed the dLGN from the dorsomedial tip to the ventrolateral end, was selected using the straight line selection tool of ImageJ. We divided the box into 1×40-pixel sections, with each section spanning the width of the box. We then took the mean intensity of each section. To normalize the mean signal intensities, they were divided by the average signal intensity in the monocular segment (200 pixels long by 40 pixels wide). The resultant signal intensities of each dLGN were plotted against the distance from the dorsomedial tip of the dLGN (Figure S2, arrow).

To quantify the expansion of ipsilateral retinogeniculate projections along the O-I and DM-VL axes (Figure 5A), small CTB-positive areas whose sizes were equal to or less than 150 pixels were removed from binarized images [40]. The remaining CTB-positive areas whose sizes were more than 150 pixels (hereafter referred to as ipsilateral patches) [39], [40] were used for further analyses (Figure 1A). To measure the length of the ipsilateral patch along the DM-VL axis, line *r′* was drawn in parallel to line *r*, which connected the dorsomedial and ventrolateral tip of the dLGN, so that the length of the ipsilateral patch (*Q_1_Q_2_*) was maximum (Figure S5A). The lengths of the dLGN (*D′V′*) and of the ipsilateral patch (*Q_1_Q_2_*) along the DM-VL axis were measured on line *r′*. The normalized length of the ipsilateral patch along the DM-VL axis was calculated as follows: *Q_1_Q_2_*/*D′V*′×100. As for the length of the ipsilateral patch along the O-I axis, line *s* was drawn perpendicular to the surface of the dLGN so that the line ran through the center of mass of ipsilateral projections (point *C*) (Figure S5B). The lengths of the dLGN (*LM*) and of the ipsilateral patch (*P_1_P_2_*) along the O-I axis were measured on line *s*. The normalized length of the ipsilateral patch along the O-I axis was calculated as follows: *P_1_P_2_*/*LM*×100.

For quantifying VGLUT1 immunoreactivity, the dLGN ipsilateral to the CTB-injected eye was photographed and examined as follows. First, a rectangular area, which was 40 pixels wide and which traversed the dLGN from the dorsomedial tip to the ventrolateral end through the central part of the CTB-positive ipsilateral projection, was selected (Figure S4D). After the background fluorescence intensity, which was the mean fluorescence intensity in the corresponding area of the section stained without primary antibody, was subtracted, mean fluorescence intensities of each 40 pixels were calculated. Because fluorescence intensities varied among experiments, the resultant fluorescence intensities were normalized by dividing them by the average fluorescence intensity of the rectangular area.

### Statistical analyses

For comparisons between 2 groups, the standard deviations (S.D.) of 2 groups were compared using the *F*-test. If S.D. were not significantly different (*P*≥0.05, *F*-test), the averages of the 2 groups were compared using the unpaired Student's *t*-test. If S.D. were significantly different (*P*<0.05, *F*-test), the averages of the 2 groups were compared using the unpaired Welch's *t*-test. For comparisons among 3 groups or more, the Tukey-Kramer test was applied.

## Supporting Information

Figure S1 Effects of ME on the sizes of the CTB-positive area and of the dLGN. ME was performed at P10, and CTB was injected into the other eye. Coronal sections of the dLGN ipsilateral to the CTB-injected eye were prepared at P35-P37. (A) The sizes of the CTB-positive areas in the dLGN. The CTB-positive areas were significantly larger in ME-treated mice (n = 5) than in control mice (n = 3). (*) *P*<0.05, unpaired Student's *t*-test. Error bars represent S.D. (B) The sizes of the dLGNs were significantly smaller in ME-treated mice (n = 5) than in control mice (n = 3). (**) *P*<0.01, unpaired Student's *t*-test. Error bars represent S.D.
(10.92 MB TIF)

Figure S2 Area used for measuring CTB signal intensities within the dLGN contralateral to the CTB-injected eye. CTB signal intensities in a rectangular area (white box), which traversed the dLGN from the dorsomedial tip (arrow) to the ventrolateral end, were measured (see Materials and Methods for details). The average signal intensities were plotted against the distance from the dorsomedial tip (arrow) of the dLGN. The lower panel is the same as Figure 2C. Scale bar represents 200 µm.
(1.10 MB TIF)

Figure S3 VGLUT2-positive areas in the dLGN largely coincide with the CTB-positive areas in ME-treated animals. ME was performed at P10 (C, D) or P34 (A, B), and CTB was injected into the other eye. Coronal sections of 30 µm thickness were prepared 25–27 days later. The distribution patterns of VGLUT2 immunoreactivity (green) and CTB (red) in the dLGN ipsilateral to the remaining eye are shown. The distribution of VGLUT2 immunoreactivity largely coincided with that of CTB-positive RGC axons. It should be noted that some VGLUT2-immunoreactive puncta did not colocalize with CTB in the outer dLGN (arrowheads), presumably because they were derived from neurons in the superior colliculus. White lines show the boundaries of the dLGN. Scale bars represent 200 µm.
(4.27 MB TIF)

Figure S4 Effect of ME on the distribution of VGLUT1 in the dLGN. ME was performed at P10, and CTB was injected into the other eye. Coronal sections of 30 µm thickness were prepared from the dLGN ipsilateral to the CTB-injected eye at P35–P37. (A, B) VGLUT1 immunoreactivity in the dLGN in ME-treated animals (A) and in control animals (B). White lines show the boundaries of the dLGN. Scale bars represent 200 µm. (C) A high magnification image of VGLUT1 staining in the dLGN. Scale bar represents 10 µm. (D) Quantification of VGLUT1 signal intensities within the dLGN. VGLUT1 signal intensities in a rectangular area (green box) in the dLGN were plotted against the distance from the dorsomedial tip of the dLGN (see Materials and Methods for details). Normalized fluorescence intensities of sections from ME-treated (red, n = 6) and control (blue, n = 3) animals are shown. Each line represents data derived from one dLGN section. Scale bars represent 200 µm.
(2.85 MB TIF)

Figure S5 Methods for analyzing ME-induced rearrangement along the DM-VL and O-I axes. (A) A diagram of the dLGN illustrating the quantification along the DM-VL axis. Line *r* (black line) was drawn to connect the most dorsomedial point (*D*) and the most ventrolateral point (*V*) that touched the optic tract in the dLGN. Line *r′* (red line) was drawn in parallel to the line *r* so that the length of the ipsilateral patch along the DM-VL axis (*Q_1_Q_2_*) was maximum. The length of *Q_1_Q_2_* and that of *D′V′* were used as the length of the ipsilateral patch (gray) and that of the dLGN along the DM-VL axis, respectively. (B) A diagram of the dLGN illustrating the quantification along the O-I axis. Point *C* was the center of mass of ipsilateral projections. Line *s* (red line) was drawn perpendicular to the surface of the dLGN so that line *s* ran through point *C*. The length of *P_1_P_2_* and that of *LM* were used as the length of the ipsilateral patch and that of the dLGN along the O-I axis, respectively.
(0.32 MB TIF)

Figure S6 The effects of TTX on the expression of immediate early genes in V1. (A) Experimental procedure of TTX treatment. After TTX was injected into the left eye at P16, mouse pups were put in darkness overnight. Twenty-four hours later, they were stimulated with light for 30–60 min, and the right visual cortex was subjected to *in situ* hybridization analyses. The monocular zones in the right V1 (blue box) are shown in (B) and (C). (B) The expression of *Arc* in the monocular zone of V1. After treated with either TTX, saline or ME, mice were stimulated with or without light. (C) The expression of *c-fos* in the monocular zone of V1. After treated with either TTX, saline or ME, mice were stimulated with or without light. Note that the expression of *Arc* and that of *c-fos* were markedly suppressed in TTX-treated mice compared with those in saline-treated mice. Scale bars represent 250 µm.
(2.30 MB TIF)
