# Supplementary figures and images for: Rearrangement of Retinogeniculate Projection Patterns after Eye-Specific Segregation in Mice
Source: PLoS One. 2010 Jun 8;5(6):e11001. doi: 10.1371/journal.pone.0011001 (PMC2882329; doi:10.1371/journal.pone.0011001)

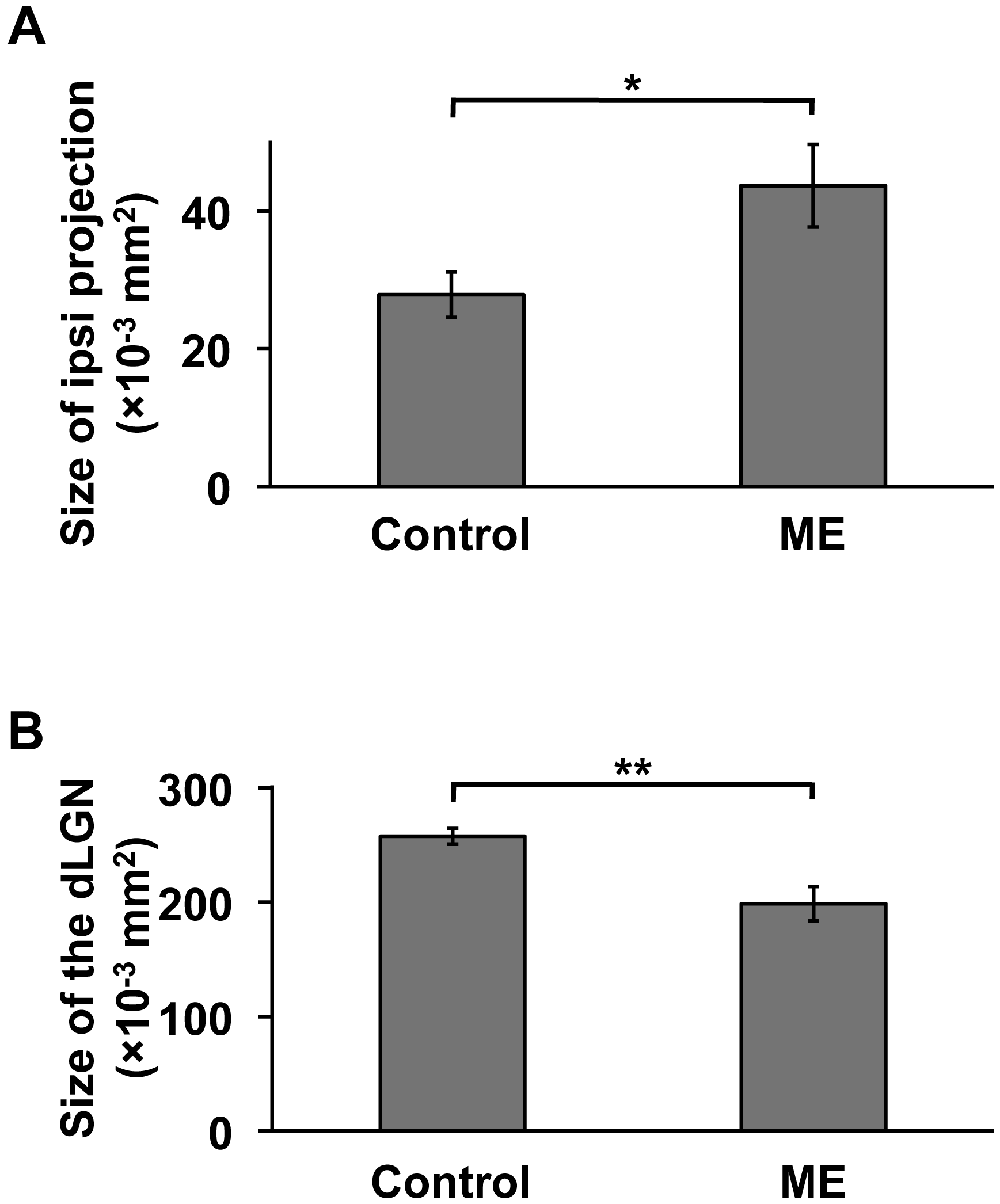

Supplement: Figure S1 — Effects of ME on the sizes of the CTB-positive area and of the dLGN. ME was performed at P10, and CTB was injected into the other eye. Coronal sections of the dLGN ipsilateral to the CTB-injected eye were prepared at P35-P37. (A) The sizes of the CTB-positive areas in the dLGN. The CTB-positive areas were significantly larger in ME-treated mice (n = 5) than in control mice (n = 3). (*) P<0.05, unpaired Student's t-test. Error bars represent S.D. (B) The sizes of the dLGNs were significantly smaller in ME-treated mice (n = 5) than in control mice (n = 3). (**) P<0.01, unpaired Student's t-test. Error bars represent S.D. (10.92 MB TIF) [file pone.0011001.s001.tif]

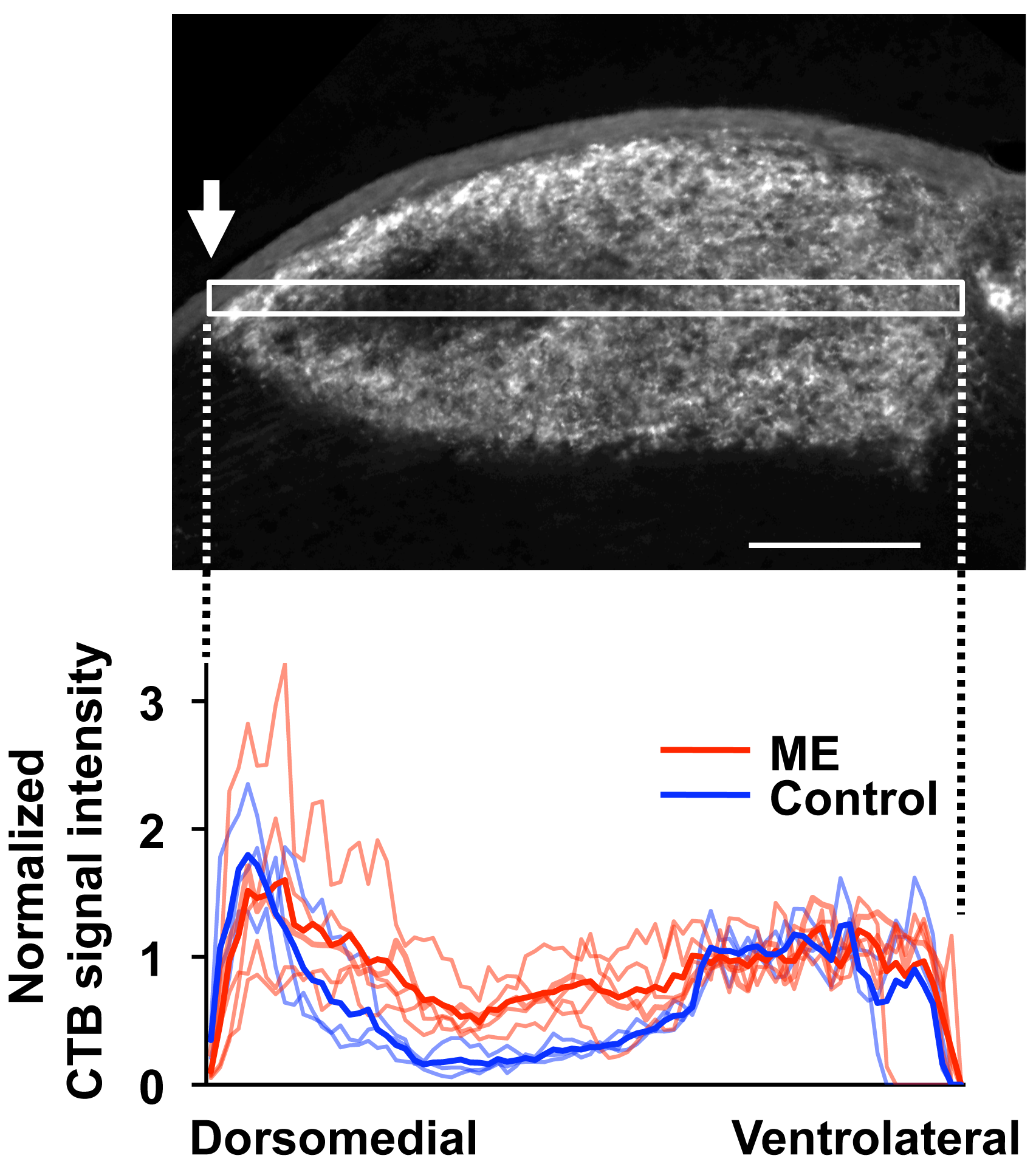

Supplement: Figure S2 — Area used for measuring CTB signal intensities within the dLGN contralateral to the CTB-injected eye. CTB signal intensities in a rectangular area (white box), which traversed the dLGN from the dorsomedial tip (arrow) to the ventrolateral end, were measured (see Materials and Methods for details). The average signal intensities were plotted against the distance from the dorsomedial tip (arrow) of the dLGN. The lower panel is the same as Figure 2C. Scale bar represents 200 µm. (1.10 MB TIF) [file pone.0011001.s002.tif]

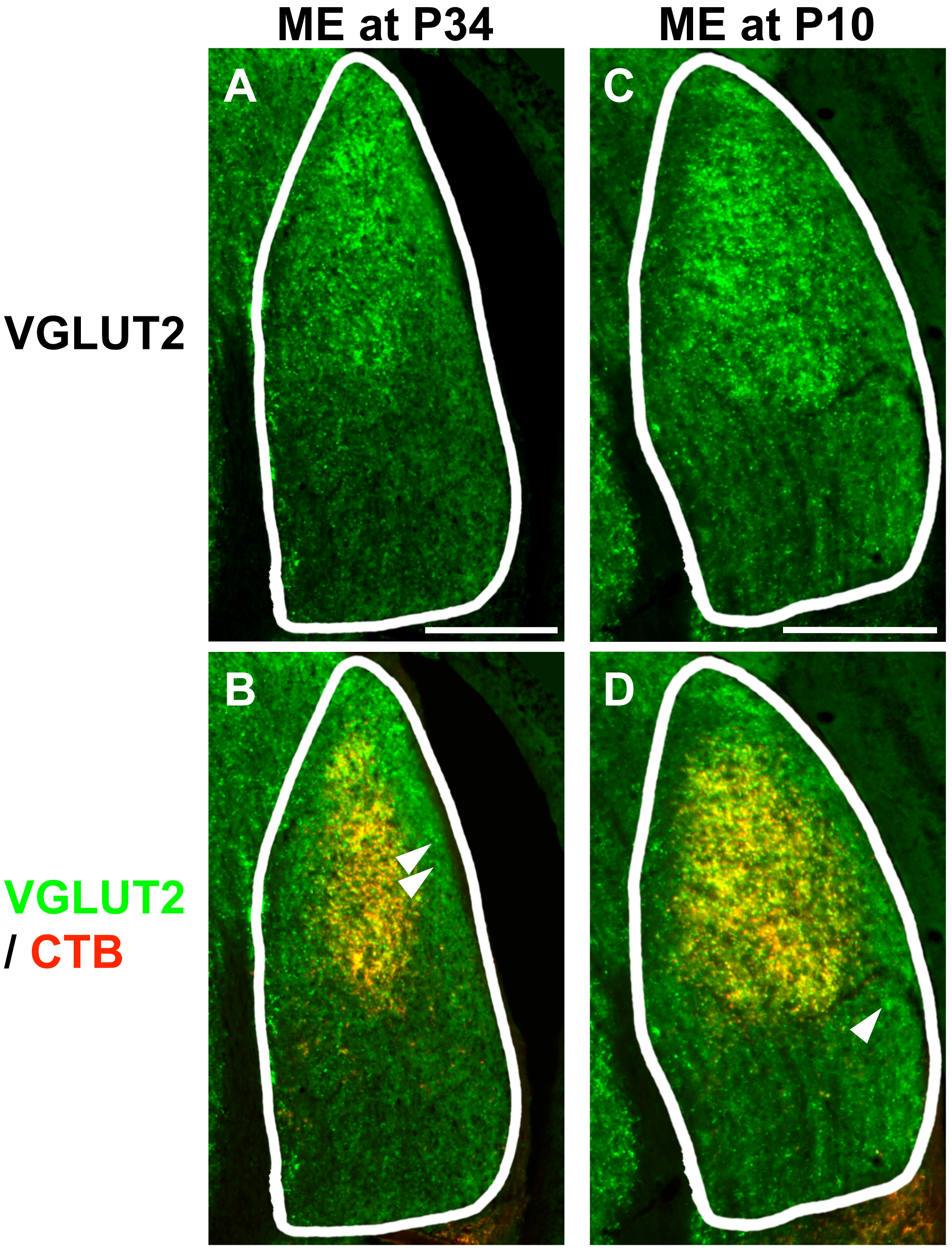

Supplement: Figure S3 — VGLUT2-positive areas in the dLGN largely coincide with the CTB-positive areas in ME-treated animals. ME was performed at P10 (C, D) or P34 (A, B), and CTB was injected into the other eye. Coronal sections of 30 µm thickness were prepared 25–27 days later. The distribution patterns of VGLUT2 immunoreactivity (green) and CTB (red) in the dLGN ipsilateral to the remaining eye are shown. The distribution of VGLUT2 immunoreactivity largely coincided with that of CTB-positive RGC axons. It should be noted that some VGLUT2-immunoreactive puncta did not colocalize with CTB in the outer dLGN (arrowheads), presumably because they were derived from neurons in the superior colliculus. White lines show the boundaries of the dLGN. Scale bars represent 200 µm. (4.27 MB TIF) [file pone.0011001.s003.tif]

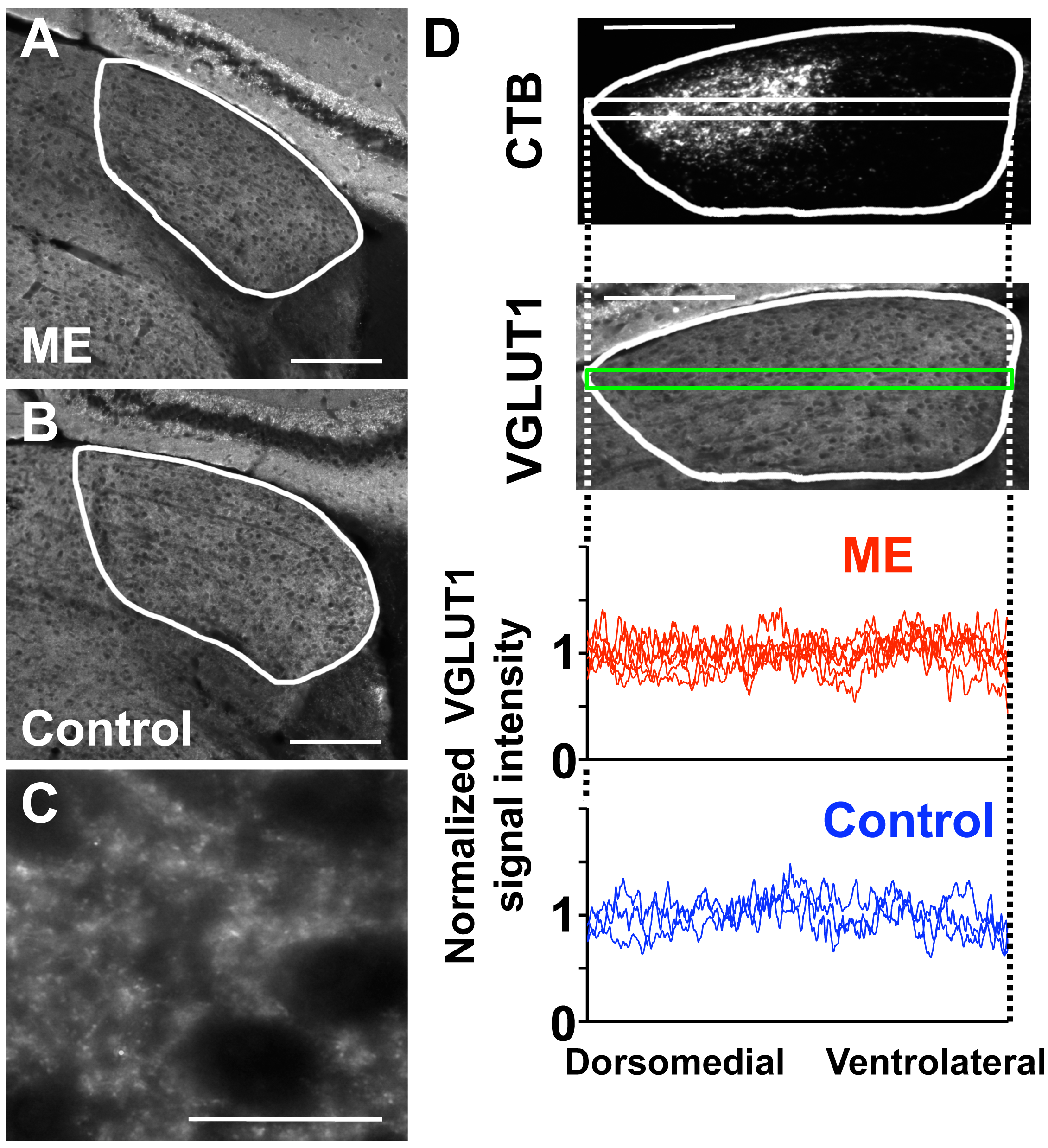

Supplement: Figure S4 — Effect of ME on the distribution of VGLUT1 in the dLGN. ME was performed at P10, and CTB was injected into the other eye. Coronal sections of 30 µm thickness were prepared from the dLGN ipsilateral to the CTB-injected eye at P35–P37. (A, B) VGLUT1 immunoreactivity in the dLGN in ME-treated animals (A) and in control animals (B). White lines show the boundaries of the dLGN. Scale bars represent 200 µm. (C) A high magnification image of VGLUT1 staining in the dLGN. Scale bar represents 10 µm. (D) Quantification of VGLUT1 signal intensities within the dLGN. VGLUT1 signal intensities in a rectangular area (green box) in the dLGN were plotted against the distance from the dorsomedial tip of the dLGN (see Materials and Methods for details). Normalized fluorescence intensities of sections from ME-treated (red, n = 6) and control (blue, n = 3) animals are shown. Each line represents data derived from one dLGN section. Scale bars represent 200 µm. (2.85 MB TIF) [file pone.0011001.s004.tif]

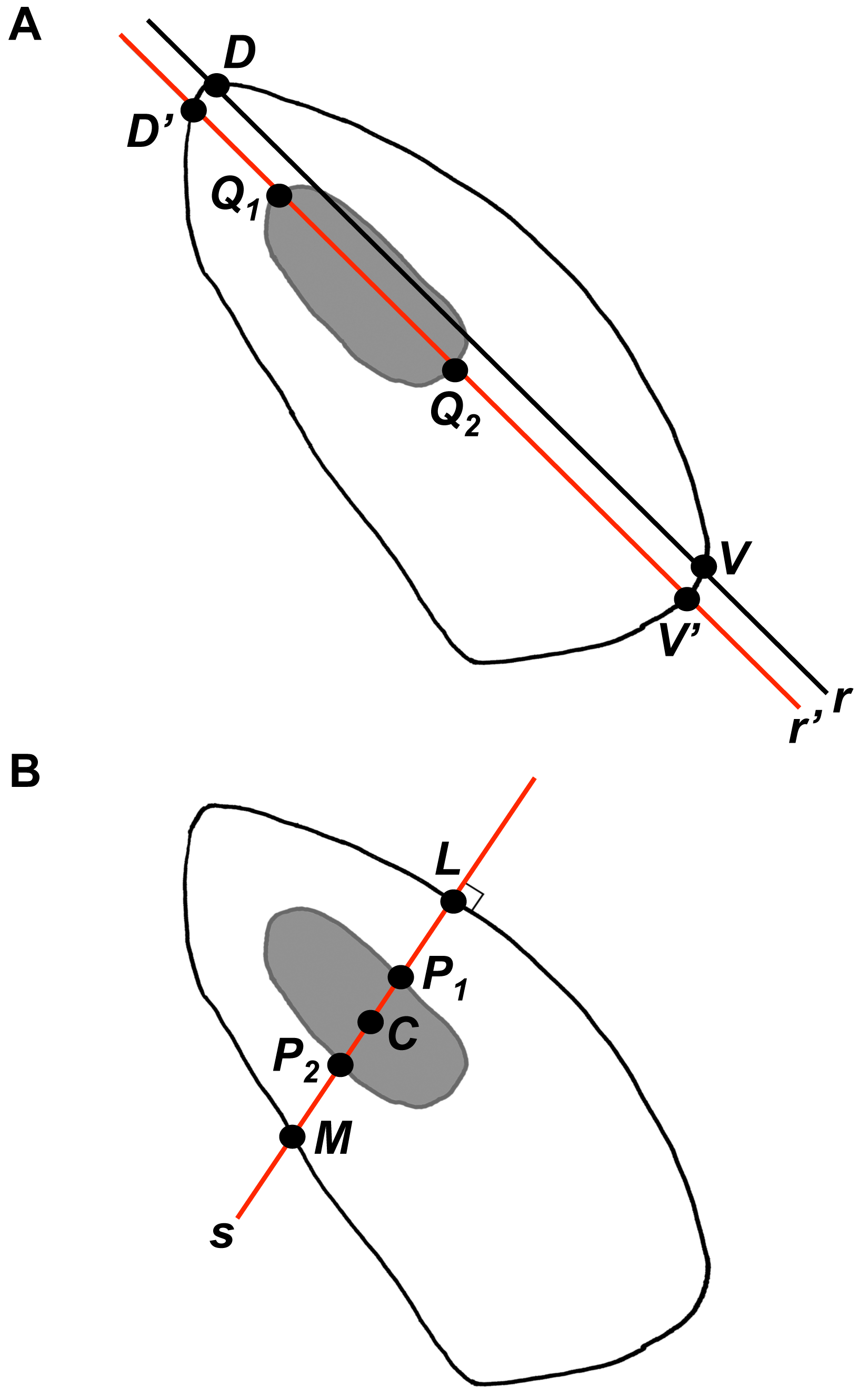

Supplement: Figure S5 — Methods for analyzing ME-induced rearrangement along the DM-VL and O-I axes. (A) A diagram of the dLGN illustrating the quantification along the DM-VL axis. Line r (black line) was drawn to connect the most dorsomedial point (D) and the most ventrolateral point (V) that touched the optic tract in the dLGN. Line r′ (red line) was drawn in parallel to the line r so that the length of the ipsilateral patch along the DM-VL axis (Q1Q2) was maximum. The length of Q1Q2 and that of D′V′ were used as the length of the ipsilateral patch (gray) and that of the dLGN along the DM-VL axis, respectively. (B) A diagram of the dLGN illustrating the quantification along the O-I axis. Point C was the center of mass of ipsilateral projections. Line s (red line) was drawn perpendicular to the surface of the dLGN so that line s ran through point C. The length of P1P2 and that of LM were used as the length of the ipsilateral patch and that of the dLGN along the O-I axis, respectively. (0.32 MB TIF) [file pone.0011001.s005.tif]

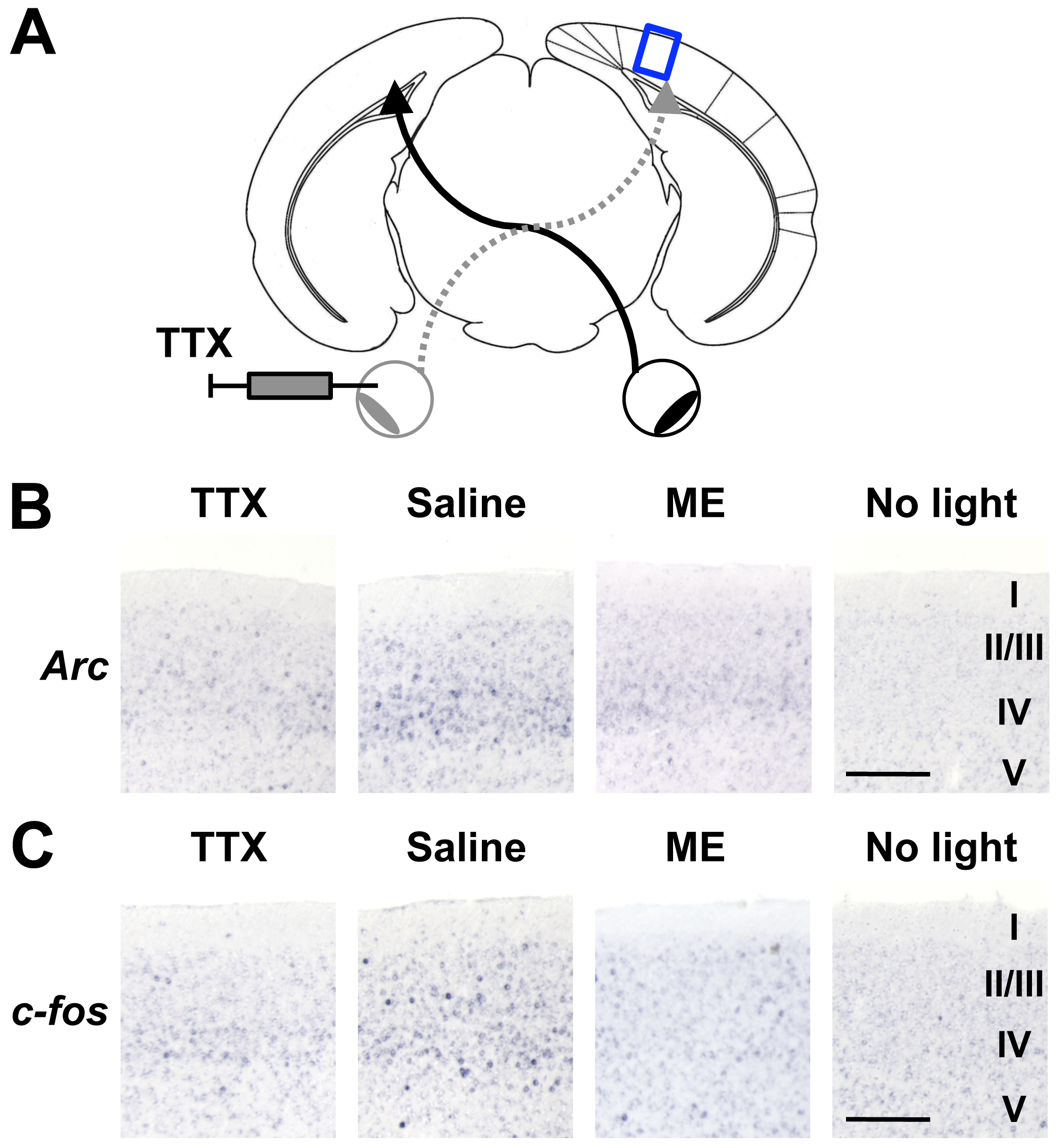

Supplement: Figure S6 — The effects of TTX on the expression of immediate early genes in V1. (A) Experimental procedure of TTX treatment. After TTX was injected into the left eye at P16, mouse pups were put in darkness overnight. Twenty-four hours later, they were stimulated with light for 30–60 min, and the right visual cortex was subjected to in situ hybridization analyses. The monocular zones in the right V1 (blue box) are shown in (B) and (C). (B) The expression of Arc in the monocular zone of V1. After treated with either TTX, saline or ME, mice were stimulated with or without light. (C) The expression of c-fos in the monocular zone of V1. After treated with either TTX, saline or ME, mice were stimulated with or without light. Note that the expression of Arc and that of c-fos were markedly suppressed in TTX-treated mice compared with those in saline-treated mice. Scale bars represent 250 µm. (2.30 MB TIF) [file pone.0011001.s006.tif]
